# Low back and neck and shoulder pain in members and non-members of adolescents’ sports clubs: the Finnish Health Promoting Sports Club (FHPSC) study

**DOI:** 10.1186/s12891-016-1114-8

**Published:** 2016-07-01

**Authors:** M. Rossi, K. Pasanen, S. Kokko, L. Alanko, O. J. Heinonen, R. Korpelainen, K. Savonen, H. Selänne, T. Vasankari, L. Kannas, U. Kujala, J. Villberg, J. Parkkari

**Affiliations:** Tampere Research Center of Sports Medicine, P.O. Box 30, FI-33501 Tampere, Finland; Clinic for Sports and Exercise Medicine, Alppikatu 2, FI-00530 Helsinki, Finland; Paavo Nurmi Centre & Department of Health and Physical Activity, University of Turku, Kiinamyllynkatu 10, FI-20520 Turku, Finland; Department of Sports and Exercise Clinic, Oulu Deaconess Institute, Albertinkatu 18, FI-90100 Oulu, Finland; Department of Health Sciences, University of Jyväskylä, P.O. Box 35, FI-40014 Jyväskylä, Finland; Kuopio Research Institute of Exercise Medicine, Haapaniementie 16, FI-70100 Kuopio, Finland; LIKES Foundation for Sports and Health Sciences and Mehiläinen Physical Activity Clinic, P.O. Box 35, FI-40720 Jyväskylä, Finland; UKK Institute for Health Promotion Research, P.O. Box 30, FI-33501 Tampere, Finland; University of Oulu, Center for Life Course Health Research, Oulu, Finland; Medical Research Center, University of Oulu and University Hospital of Oulu, Oulu, Finland

**Keywords:** Neck and shoulder pain, Low back pain, Adolescence, Sports club participation, Prevalence

## Abstract

**Background:**

The objective of this study was to investigate the prevalence of self-reported low back pain (LBP) and neck and shoulder pain (NSP), and the related factors in members and non-members of adolescents’ sports clubs.

**Methods:**

This cross-sectional study was based on surveys of 14–16-year-olds as a part of the Finnish Health Promoting Sports Club (FHPSC) Study. The surveys on self-reported health behaviours, injuries, and musculoskeletal health were conducted among sports club members (*n* = 962) and non-members (*n* = 675). Binary logistic regression analysis was applied to study the associations between dependent variables of LBP and NSP, and the independent factors.

**Results:**

The prevalence of LBP during the preceding 3 months was 35.0 % in girls and 24.5 % in boys (*p* < 0.05 for sex difference). The prevalence of NSP was 55.9 % in girls and 27.3 % in boys (*p* < 0.001 for sex difference). Being a sports club member increased the odds for LBP in boys (odds ratio [OR] 2.35, 95 % CI 1.48–3.72). On the other hand, sports club participation was associated with lower odds of frequent NSP in girls (OR 0.52, 95 % CI 0.33–0.82). No associations were found between other leisure-time physical activity and LBP or NSP. Higher screen time (computer games, TV/DVD, phone, Internet) during leisure-time increased the odds of NSP in boys and LBP in boys and girls.

**Conclusions:**

In this study, self-reported LBP and NSP were already relatively common among adolescents. Girls have a higher risk for reporting LBP and NSP. Measures that are more effective in the prevention of LBP in male sports club members are needed. Excessive screen time is weakly associated with LBP and NSP, which should be taken into account in health promotion among adolescents.

**Electronic supplementary material:**

The online version of this article (doi:10.1186/s12891-016-1114-8) contains supplementary material, which is available to authorized users.

## Background

Back problems are a major public health problem. In Finland in 2013, back diseases were responsible for a sickness benefit expenditure of approximately 118 million euros, and they caused over two million days of covered illness [1]. Backache itself caused approximately 787,000 covered days of illness [1]. Low back pain (LBP) is relatively common already among adolescents [2]. Neck and shoulder pain (NSP) has been studied less, especially among adolescent athletes [2–4], but the prevalence of NSP seems to have increased during the 21^st^ century [3]. The prevalence of LBP increases with age [5, 6]. Among 15–16-years-olds, LBP prevalence has been reported to be 32 % in boys and 45 % in girls [7]. Five per cent of those aged 15–16 years (*n* = 7344) sought medical assistance due to their LBP symptoms [7]. LBP in adolescence has a tendency of increasing the probability of LBP also in adulthood [8], and it is commonly concurrent with other musculoskeletal pain [9]. Therefore, it is important to identify risk populations and to effect the early prevention of LBP and NSP. Some studies have already investigated the differences in LBP between adolescent athletes and non-athletes [10, 11]. Physical activity as a risk factor has been studied previously [10, 12–14]. However, the results remain inconclusive.

This study is a part of a multidisciplinary and multi-institutional study (the Finnish Health Promoting Sports Club (FHPSC)) [15] where the overall aim is to investigate the effects of sports club participation and the activity of health promotion within sports clubs on adolescent health. Therefore, the specific objectives of this study were to determine the prevalence, frequency, and severity of LBP and NSP in the 14–16-year-old population. We also explored the associations between LBP and NSP with the health and health behaviour of adolescents, paying special attention to participation in organized sports (sports club membership).

## Methods

This study is part of the Finnish Health Promoting Sports Club (FHPSC) study conducted in Finland by the University of Jyväskylä in conjunction with six sports medicine centres and the UKK institute [15]. This cross-sectional study was based on surveys among 14–16-year-olds, and it was carried out in accordance with the Declaration of Helsinki. The adolescents were notified that they have a right to refuse to participate and withdraw their consent later without giving a reason. A written consent from both a guardian and the adolescent him/herself for the pre-participation screening were obtained for participants under the age of 16. Ethical approval was received from the Ethics Committee of Health Care District of Central Finland (record number 23U/2012). All permission papers included detailed information of the study.

### Data collection

**Fig. 1:**
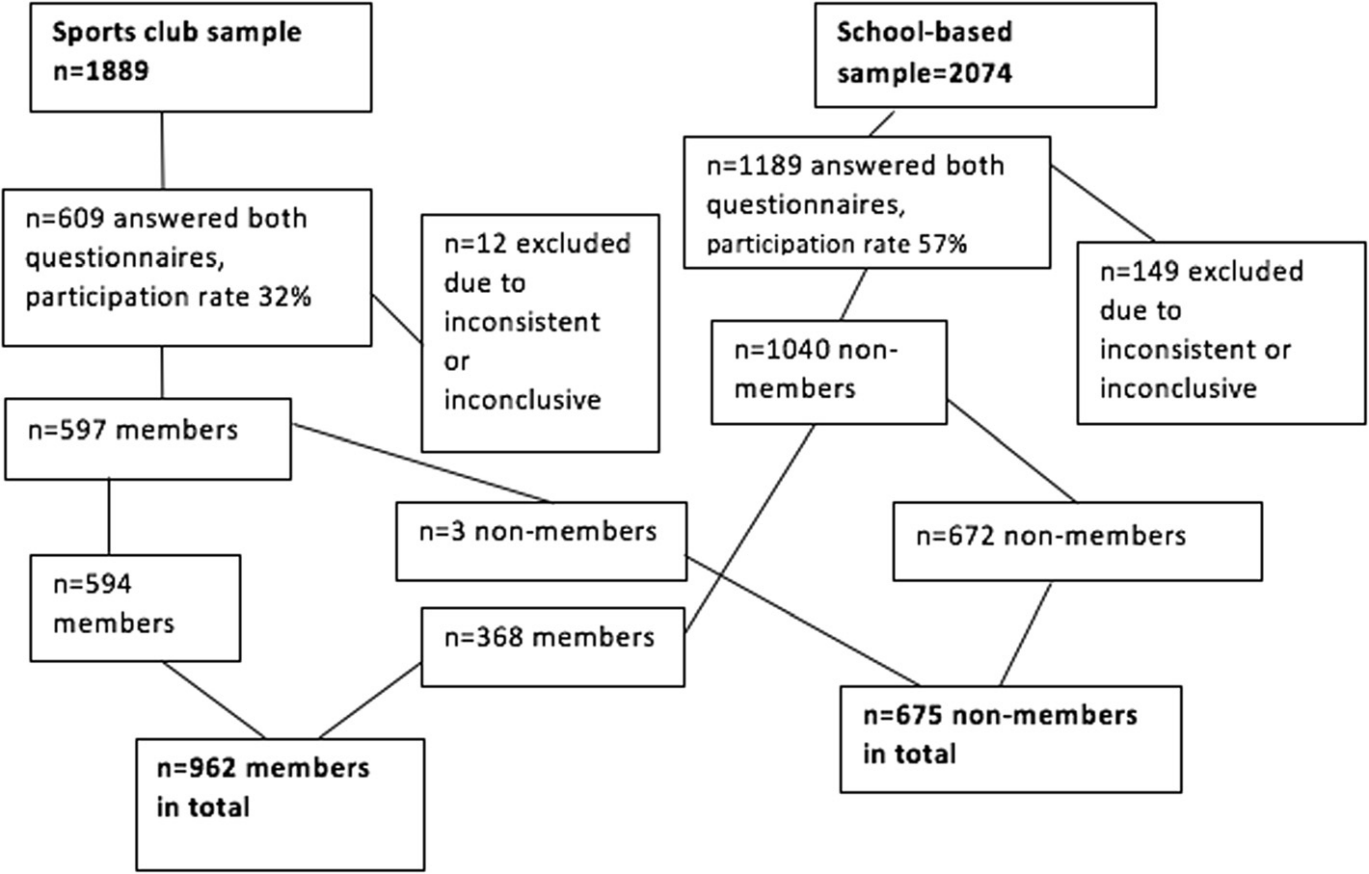
Study sample

In order to obtain a nationally representative sample of the most popular sports for youths, a total of two hundred and forty youth sports clubs from the ten most popular sports disciplines in Finland (basketball, cross-country skiing, floorball, football, gymnastics, ice-hockey, orienteering, skating, swimming, and track and field) were targeted. Twenty-four clubs were selected from each sport for the sample and 154 youth sports clubs out of 240 participated (64 %) in the FHPSC study. Data was collected in the middle of the main competition season from January to May 2013 for winter sports, and from August to December 2013 for summer sports. In total, 1889 sports club participants were invited to participate in two separate internet questionnaires (Fig. 1.). From the sports clubs 609 adolescents completed both questionnaires.

In order to compare the health behaviours and health status of youths participating in organized sports clubs (club members) to non-participating youths (non-members), the second sample included in this study was a group of secondary school children aged 14–16. The schools were collated from each district where the sports medicine centres were located, including nearby rural areas. School-based data was collected in two portions following the sports clubs’ data collection timeframe (100 schools participated). In total, 2074 pupils were asked to participate in the study during the normal school day and 1189 completed both questionnaires.

Members of the school-based sample were asked about their sports club participation (“At the moment are you a member of a sports club?” “no/yes/yes, but I don’t participate to training provided by the club”) and those who reported being members of a sports club were treated as sports club members (*n* = 368) in the following analyses. Three subjects from the sports club sample were analysed as non-members as they reported not participating in sports club activities (answered no to the question “Are you participating in sports club activities?” “yes/no”). Subjects that provided inconsistent or inconclusive concerning gender and/or age or sports club membership were excluded (*n* = 12 from sports club sample and *n* = 149 from school-based sample). In total, 962 sports club members (368 from school-based sample and 594 from sports club sample) and 675 non-members were included (*n* = 1637) (Fig. 1.).

### Surveys

Two surveys were conducted (see Additional files 1 and 2). The first focused on the health behaviours of the adolescents, including self-evaluated overall physical activity. The questions included for example: “How many hours on a regular school day you spend your time sitting with one of the following devices? (TV, video/DVD, computer, console games, tablet/phone)” and “Outside school hours: How many hours do you usually do physical activity so that you sweat and get out of breath?”. Unlike the questionnaire for non-members, the questionnaire for sports club members included some extra questions on training characteristics, such as active playing/practicing years (at least 2 times a week), training frequency, duration and number of rest days during training and competition seasons, as well as number of competitions. The second questionnaire focused on injuries and the musculoskeletal health of the adolescents. The questions used in these questionnaires were compiled from previously validated questions in other studies, like the Health Behaviour in School-aged Children (HBSC) study [16–20].

### Outcomes

The main dependent outcomes were NSP and LBP within the preceding 3 months. The questions in the questionnaire were “How often have you had the following symptoms in the preceding 3 months?” Answer options included “aches or pain in the neck and shoulders” and “aches or pain in the low back” daily, more than once a week, approximately once a week, 2–3 times a month, approximately once a month, and less than once a month or not at all. Two dependent variables were formed for both LBP and NSP. These were LBP (low back pain more than once a month) and frequent LBP (low back pain at least once a week), and NSP (neck and shoulder pain more than once a month) and frequent NSP (neck and shoulder pain at least once a week). Questions that were more specifically about LBP were based on the standardized Nordic questionnaire of musculoskeletal symptoms [21]. LBP was defined as “an ache, pain, or discomfort of the lumbar region with or without radiation to one or both legs (sciatica).” The questions in the questionnaire included:

- “Have you ever experienced problems in your low back?” (area illustrated by a picture) (no/yes)
- “Have you ever had surgery because of LBP?” (no/yes)
- “Have you ever had radiating LBP?” (no/yes)
- “Have you ever had sleeping difficulties because of LBP?”(no/yes, how often?)
- “Have you had LBP during the previous 7 days?” (no/yes)
- “Have you experienced low back pain that has required consultation or treatments by a physician, physiotherapist, or chiropractor in the previous 12 months?” (no/yes)
- “How did your LBP symptoms occur?” suddenly (after an injury)/gradually (without a sudden injury)/or both
- “Have you used pain killers (NSAID) for your low back?” (no/yes)

### Statistical methods

IBM SPSS Statistics (v. 22.0) was used to carry out all analyses. Sample size was power calculated by Stata 11.0 using data of Kokko et al. [18]. Differences between the groups were assessed using crosstabs and the chi-square test (and *t*-test when appropriate). The subject characteristics are presented for girls and boys, and sports club members and non-members separately as means ± SDs and percentages. Low back pain and neck and shoulder pain prevalence are expressed as a number and percentage of members and non-members separately for girls and boys. As multilevel modelling failed to give additional information, binary logistic regression analysis was applied to study the associations between the dependent variables of LBP (low back pain) and NSP (neck and shoulder pain) and the independent factors. Binary logistic regression analyses were adjusted by age, sex, BMI, chronic diseases, smoking, and school attainment level (i.e. school grade average). The binary logistic regression analyses were conducted separately for health, health behaviour and training variables. In the analyses for the health and health behaviour the variables were entered into the model simultaneously. In the analyses for training variables separate analyses were conducted for all variables. Odds ratios are reported with 95 % confidence intervals. *P*-value, 0.05 was regarded significant.

## Results

The significant differences in background characteristics between sports club members and non-members are highlighted in Table 1. There were more girls who had already had menarche among non-members than members (97.5 % vs 92.7 %, *p* < 0.001). The use of dietary supplements and pain killers (NSAIDs) was more frequent among sports club members. They were physically more active in their leisure time than non-members and had shorter daily screen time ((mean) 4.1 vs 5.9 h/day, *p* < 0.001).

**Table 1:** Subject characteristics by sports club participation and gender

	Boys (*n* = 772)			Girls (*n* = 865)			Total (*n* = 1637)		
Variable	Member	Non-member	*P*-value^*^	Member	Non-member	*P*-value^*^	Member	Non-member	*P*-value^*^
Age, mean (SD)	15.5(1)	15.5(0)	0.643	15.5(1)	15.5(0)	0.880	15.5(1)	15.5(1)	0.839
BMI, mean (SD)	**20.9(2)**	**21.5(4)**	**<0.05**	**20.6(2)**	**21.1(4)**	**<0.05**	**20.7(2)**	**21.3(4)**	**<0.002**
Menarche,% (*n* = 882)	-	-		**92.8 %**	**97.5 %**	**<0.001**	-	-	-
Chronic disease,^a^%	30.4 %	26.4 %	0.242	30.1 %	29.4 %	0.818	30.2 %	28.1 %	0.358
Regular medication,^b^%	23.5 %	18.6 %	0.113	29.0 %	33.2 %	0.189	26.2 %	27.1 %	0.680
NSAID use, previous month,%	**59.6 %**	**46.1 %**	**<0.001**	75.0 %	73.7 %	0.655	**67.2** %	**62.2** %	**<0.05**
Special diet,^c^%	8.0 %	5.7 %	0.245	17.2 %	18.0 %	0.754	12.5 %	12.9 %	0.804
Dietary supplements use,^d^%	**67.3** %	**36.8** %	**<0.001**	**70.1** %	**57.2** %	**<0.001**	**68.7** %	**48.7** %	**<0.001**
No Smoking, %	**94.5** %	**81.4** %	**<0.001**	**92.6** %	**73.9** %	**<0.001**	**93.6** %	**77.0** %	**<0.001**
Screen time,^e^ mean (SD)	**4.6(4)**	**6.4(5)**	**<0.001**	**3.6(2)**	**5.6(5)**	**<0.001**	**4.1(3)**	**5.9(5)**	**<0.001**
Leisure time PA, ^f, g^ %			**<0.001**			**<0.001**			**<0.001**
Approx. <30 min/week	**0.8 %**	**15.4 %**		**0.9 %**	**18.5 %**		**0.8 %**	**17.2 %**	
Approx. 1–3 h/week	**16.3 %**	**53.9 %**		**14.9 %**	**56.9 %**		**15.6 %**	**55.6 %**	
Approx. 4–6 h/week or more	**82.9 %**	**30.7 %**		**84.2 %**	**24.6 %**		**83.5 %**	**27.2 %**	

Statistically significant findings are indicated in bold

^*^ *p*-value for difference between members and non-members of sports clubs

^a^Allergy, asthma, diabetes, epilepsy, heart condition, etc.

^b^Contraceptives or other hormonal medication, allergy, asthma, insulin, epilepsy, or heart or blood pressure medication

^c^Vegetarian, low carb, lactose free, dairy free, gluten free, or other special diet

^d^For example, vitamins, protein supplements, amino acid supplements, creatine

^e^TV, computer, computer/console games, phone, tablet use

^f^Boys *n* = 770, girls *n* = 863, total *n* = 1633

^g^Intensity: breathlessness and sweating

### Low back pain

The prevalence of self-reported LBP during the preceding 3 months was 35.0 % in all girls (*n* = 865) and 24.5 % in all boys (*n* = 772) (*p* < 0.001 for sex difference) girls being more likely to have frequent LBP than boys (OR 2.33 95 % CI 1.58–3.45). No differences between sports club members and non-members were found in girls for LBP (Table 2). However, the prevalence of LBP during the preceding 3 months was significantly higher in male sports club members than in non-members (28.1 % vs 18.1 %, *p* < 0.02) (Table 2).

**Table 2:** Prevalence of LBP in members and non-members of sports clubs

		Boys (*n* = 772)					Girls (*n* = 865)				
Variable	Category	Member		Non-member		*P*-value^*^	Member		Non-member		*P*-value^*^
		*n*	%	*n*	%		*n*	%	*n*	%	
Lifetime prevalence	Yes	**259**	**52.7**	**122**	**43.4**	**<0.02**	284	60.3	246	62.4	0.520
	No	**232**	**47.3**	**159**	**56.6**		187	39.7	148	37.6	
LBP^a^	Yes	**138**	**28.1**	**51**	**18.1**	**0.02**	160	34.0	143	36.3	0.475
	No	**353**	**71.9**	**230**	**81.9**		311	66.0	251	63.7	
Frequent LBP^b^	Yes	30	6.1	12	4.3	0.278	51	10.8	44	11.2	0.874
	No	461	93.9	269	95.7		420	89.2	350	88.8	
LBP during the last seven days^c^	Yes	100	38.6	39	32.0	0.209	123	43.3	110	44.7	0.745
	No	159	61.4	83	68.0		161	56.7	136	55.3	

Statistically significant results are indicated in bold

^*^ *p*-value for difference between members and non-members of sports clubs

^a^LBP more than once a month

^b^ LBP at least once a week

^c^Boys *n* = 381, girls *n* = 530

Among boys, sports club members sought medical assistance due to their LBP significantly more often than non-members did (25.9 % vs 5.7 % respectively, *p* < 0.001). They also used significantly more NSAIDs due to LBP (Table 3). Among girls, non-members had more sleeping difficulties due to LBP compared to members (11.6 % vs 17.9 %, *p* < 0.05) (Table 3). However, LBP that radiated to the lower extremities was more common in female sports club members than in non-members (23.2 % vs 15.0 %, *p* < 0.05).

**Table 3:** Characteristics of LBP in members and non-members of sports clubs

		Boys (*n* = 381)					Girls (*n* = 530)				
Variables	Category	Member		Non-member		*P*-value^*^	Member		Non-member		*P*-value^*^
		*n*	%	*n*	%		*n*	%	*n*	%	
LBP that has demanded medical assistance in the previous 12 months^a^	Yes	**67**	**25.9**	**7**	**5.7**	**<0.001**	47	16.5	31	12.6	0.201
	No	**192**	**74.1**	**115**	**94.3**		237	83.5	215	87.4	
NSAID use due to LBP symptoms	Yes	**99**	**38.2**	**25**	**20.5**	**<0.002**	111	39.1	112	45.5	0.134
	No	**160**	**61.8**	**97**	**79.5**		173	60.9	134	54.5	
Sleeping difficulties due to LBP	Yes	13	5.0	7	5.7	0.769	**33**	**11.6**	**44**	**17.9**	**<0.05**
	No	246	95.0	115	94.3		**251**	**88.4**	**202**	**82.1**	
Radiating LBP^b^	Yes	57	22.0	19	15.6	0.143	**66**	**23.2**	**37**	**15.0**	**<0.02**
	No	202	78.0	103	84.4		**218**	**76.8**	**209**	**85.0**	
Operation due to your LBP	Yes	2	0.8	0	0.0	0.330	1	0.4	1	0.4	0.919
	No	257	99.2	122	100.0		283	99.6	245	99.6	
LBP origin						0.413					0.653
	Acute^c^	46	17.8	17	13.9		23	8.1	22	8.9	
	Overuse^d^	185	71.4	95	77.9		239	84.2	200	81.3	
	Both	28	10.8	10	8.2		22	7.7	24	9.8	

Statistically significant results are indicated in bold

^*^ *p*-value for difference between members and non-members of sports clubs

^a^ From a physician, physiotherapist, or chiropractor

^b^LBP that radiates to the lower extremities (buttocks, thigh, knee, lower leg, or foot)

^c^ After injury to low back

^d^ Slowly without injury

### Neck and shoulder pain

The prevalence of self-reported NSP was higher in girls (52.9 %) than in boys (27.3 %) (*p* < 0.001 for sex difference). In addition, the prevalence of frequent NSP was higher in girls than in boys (19.8 % vs 5.4 %, *p* < 0.001 for sex difference). Girls were more likely to have frequent NSP than boys (OR 4.44 95 % CI 3.08–6.40). As shown in Table 4, among girls, non-members had a higher prevalence of NSP than sports club members (59.9 % vs 47.1 %, *p* < 0.001). The prevalence of frequent NSP during the preceding 3 months was higher in non-members for both girls and boys (Table 4).

**Table 4:** Prevalence of NSP in members and non-members of sports clubs

		Boys (*n* = 772)					Girls (*n* = 865)				
Variables	Category	Member		Non-member		*P*-value^*^	Member		Non-member		*P*-value^*^
		*n*	%	*n*	%		*n*	%	*n*	%	
NSP^a^	Yes	130	26.5	81	28.8	0.481	**222**	**47.1**	**236**	**59.9**	**<0.001**
	No	361	73.5	200	71.2		**249**	**52.9**	**158**	**40.1**	
Frequent NSP^b^	Yes	**19**	**3.9**	**23**	**8.2**	**<0.02**	**67**	**14.2**	**104**	**26.4**	**<0.001**
	No	**472**	**96.1**	**258**	**91.8**		**404**	**85.8**	**290**	**73.6**	

Statistically significant results are indicated in bold

^*^ *p*-value for difference between members and non-members of sports clubs

^a^NSP more than once a month

^b^NSP at least once a week

### Risk factors for low back pain

Adjusted odds ratios regarding health (Table 5), health behaviour (Table 6), and training characteristics (Table 7) are shown in the tables. LBP was associated with reporting neck, thoracic spine, and lower limb pain in boys and girls, and it was also associated with upper limb pain in boys. Higher screen time, as calculated per additional hour of screen time (computer games, TV/DVD, phone, Internet) during leisure time, increased the odds slightly for LBP in boys (OR 1.07, 95 % CI 1.01–1.12) and girls (OR 1.06, 95 % CI 1.01–1.10, Table 6). For girls, screen time exceeding 4 h/day increased the odds for LBP by 1.46 (95 % CI 1.08–1.95). Associations were not found between leisure-time physical activity and LBP or frequent LBP (Table 6). However, in boys, sports club membership was associated with LBP (OR 2.35, 95 % CI 1.48–3.72). Furthermore, the odds for frequent LBP was higher in male sports club members than in non-members (OR 2.73 95 % CI 1.17–6.34) (Table 6). LBP was associated with smoking in both boys and girls, and with alcohol use in boys (Table 6).

**Table 5:** Associations between LBP and health variables in 14 to 16 year old Finnish adolescents

		LBP^a^				Frequent LBP^b^			
Variables	Category	Boys (*n* = 768)		Girls (*n* = 856)		Boys (*n* = 768)		Girls (*n* = 856)	
		OR^c^	95 % CI	OR^c^	95 % CI	OR^c^	95 % CI	OR^c^	95 % CI
Chronic diseases^d^	No	1 (referent)		1 (referent)		1 (referent)		1 (referent)	
	Yes	0.71	(0.44–1.13)	1.36	(0.95–1.96)	0.72	(0.34–1.55)	1.38	(0.84–2.25)
BMI		1.04	(0.97–1.11)	0.96	(0.91–1.02)	1.14	(0.44–2.99)	1.01	(0.94–1.09)
Neck pain	No	1 (referent)		1 (referent)		1 (referent)		1 (referent)	
	Yes^e^	**1.83**	(**1.13**–**2.96**)	**2.13**	(**1.47**–**3.09**)	1.65	(0.72–3.77)	1.74	(0.91–3.33)
Thoracic spine pain	No	1 (referent)		1 (referent)		1 (referent)		1 (referent)	
	Yes^e^	**9.39**	(**5.39**–**16.34**)	**6.31**	(**4.15**–**9.59**)	**2.88**	**(1.22–6.82)**	**4.49**	(**2.61**–**7.74**)
Upper limb pain	No	1 (referent)		1 (referent)		1 (referent)		1 (referent)	
	Yes^e^	**1.87**	**(1.02–3.44)**	1.41	(0.90–2.12)	0.77	(0.26–2.27)	**1.95**	(**1.12**–**3.40**)
Lower limb pain	No	1 (referent)		1 (referent)		1 (referent)		1 (referent)	
	Yes^e^	**1.74**	(**1.02–2.96**)	**1.53**	(**1.03**–**2.27**)	1.52	(0.61–3.76)	1.42	(0.82–2.46)

Statistically significant results are indicated in bold

^a^LBP more than once a month during the last 3 months

^b^LBP at least once a week during the last 3 months

^c^Binary logistic regression was used and all variables were included in the same model. Analyses were adjusted by age, BMI, chronic diseases, smoking, school attainment level

^d^Allergy, asthma, diabetes, epilepsy, heart condition, etc.

^e^At least once a month

**Table 6:** Associations between LBP and health behaviour variables in 14 to 16 year old Finnish adolescents

		LBP^a^				Frequent LBP^b^			
Variables	Category	Boys (*n* = 768)		Girls (*n* = 856)		Boys (*n* = 768)		Girls (*n* = 856)	
		OR^c^	95 % CI	OR^c^	95 % CI	OR^c^	95 % CI	OR^c^	95 % CI
Screen time^d^		**1.07**	**(1.01–1.12)**	**1.06**	(**1.01–1.10**)	1.04	(0.99–1.10)	1.03	(0.99–1.09)
Leisure time PA^e^	Approx. <30 min/week	1 (referent)		1 (referent)		1 (referent)		1 (referent)	
	Approx. 1–3 h/week	1.38	(0.58–3.29)	1.31	(0.74–2.31)	2.04	(0.40–10.27)	0.79	(0.35–1.79)
	Approx.4-6 h/week or more	1.56	(0.63–3.82)	1.75	(0.95–3.23)	1.11	(0.20–6.10)	1.20	(0.50–2.85)
Sports club membership	No	1 (referent)		1 (referent)		1 (referent)		1 (referent)	
	Yes	**2.35**	(**1.48–3.72)**	0.97	(0.67–1.42)	**2.73**	**(1.17–6.34)**	0.99	(0.56–1.76)
Use of alcohol	<1x month	1 (referent)		1 (referent)		1 (referent)		1 (referent)	
	1 x month	1.15	(0.59–2.22)	1.40	(0.77–2.55)	1.39	(0.45–4.29)	1.63	(0.76–3.50)
	≥2–3 x month	**2.25**	**(1.04–4.90)**	1.17	(0.61–2.25)	2.73	(0.87–8.60)	0.57	(0.19–1.75)
Smoking	No	1 (referent)		1 (referent)		1 (referent)		1 (referent)	
	Yes	**2.32**	(**1.29–4.19)**	**1.96**	(**1.26–3.04**)	1.42	(0.53–3.78)	1.46	(0.78–2.76)

Statistically significant results are indicated in bold

^a^LBP more than once a month during the last 3 months

^b^LBP at least once a week during the last 3 months

^c^Binary logistic regression was used and all variables were included in the same model. Analyses were adjusted by age, BMI, chronic diseases, smoking, school attainment level

^d^TV, computer, computer/console games, phone, tablet use, OR calculated per additional hour of screen time

^e^ Intensity: breathlessness and sweating

**Table 7:** Associations between LBP and training characteristics in 14 to 16 year old sports club members

	Boys		Girls	
Training Characteristics	OR^a^	95 % CI	OR^a^	95 % CI
Active playing/practicing years (boys *n* = 488, girls *n* = 465)	1.05	(0.97–1.13)	1.07	(1.00–1.14)
Training hours per week during training season (boys *n* = 486, girls *n* = 463)	**1.05**	**(1.01–1.09)**	1.01	(0.96–1.05)
Training hours per week during competition season (boys *n* = 482, girls *n* = 448)	1.03	(0.99–1.08)	1.03	(0.99–1.08)
Number of competitions/games during previous 12 months (boys *n* = 485, girls *n* = 462)	**1.01**	**(1.00–1.02)**	1.00	(0.98–1.01)
Number of rest days during training season (boys *n* = 483, girls *n* = 461)	**0.78**	**(0.65–0.94)**	0.96	(0.83–1.11)
Number of rest days during competition season (boys *n* = 480, girls *n* = 459)	**0.79**	**(0.66–0.94)**	**0.84**	**(0.72–1.00)**

Statistically significant results are indicated in bold

^a^All training variables analysed in separate models. Adjusted by age, BMI, chronic diseases, smoking, school attainment level

Among boys, the training hours during the training season, the number of competitions/games during the preceding 12 months increased the odds of having LBP as calculated per additional hour of training (Table 7). More rest days during the competition season decreased the odds of having LBP in boys and girls, and more rest days during the training season decreased the odds of having LBP in boys.

### Risk factors for neck and shoulder pain

Adjusted odds ratios of health (Table 8), health behaviour (Table 9), and training characteristics (Table 10) are shown in the tables. The odds for self-reported NSP were increased by having chronic disease(s) (OR 1.85, 95 % CI 1.23–2.80 for boys and OR 1.49, 95 % CI 1.05–2.10 for girls), and also with reporting low back, thoracic spine, and upper limb pain (Table 8).

**Table 8:** Associations between NSP and health variables in 14 to 16 year old Finnish adolescents

		NSP^a^				Frequent NSP^b^			
Variables	Category	Boys (*n* = 768)		Girls (*n* = 856)		Boys (*n* = 768)		Girls (*n* = 856)	
		OR^c^	95 % CI	OR^c^	95 % CI	OR^c^	95 % CI	OR^c^	95 % CI
Chronic diseases^d^	No	1 (referent)		1 (referent)		1 (referent)		1 (referent)	
	Yes	**1.85**	**(1.23–2.80)**	**1.49**	**(1.05–2.10)**	1.00	(0.46–2.18)	1.21	(0.81–1.79)
BMI		1.03	(0.97**–**1.10)	1.03	(0.97**–**1.08)	1.02	(0.91**–**1.15)	**1.07**	**(1.01–1.13)**
Low back pain	No	1 (referent)		1 (referent)		1 (referent)		1 (referent)	
	Yes^e^	**1.88**	**(1.16–3.03)**	**2.15**	**(1.48–3.11)**	1.84	(0.73–4.66)	**1.88**	**(1.22–2.91)**
Thoracic spine pain	No	1 (referent)		1 (referent)		1 (referent)		1 (referent)	
	Yes^e^	**3.53**	**(1.97–6.33)**	**4.87**	**(2.92–8.11)**	**4.91**	**(1.94–12.42)**	**3.87**	**(2.48–6.06)**
Upper limb pain	No	1 (referent)		1 (referent)		1 (referent)		1 (referent)	
	Yes^e^	**6.47**	**(3.73–11.23)**	**4.00**	**(2.39–6.61)**	1.00	(0.35–2.88)	**1.94**	**(1.21–3.10)**
Lower limb pain	No	1 (referent)		1 (referent)		1 (referent)		1 (referent)	
	Yes^e^	1.39	(0.83–2.33)	1.44	(0.98–2.12)	0.65	(0.23–1.88)	1.07	(0.68–1.68)

Statistically significant results are indicated in bold

^a^NSP more than once a month during the last 3 months

^b^NSP at least once a week during the last 3 months

^c^Binary logistic regression was used and all variables were included in the same model. Analyses were adjusted by age, BMI, chronic diseases, smoking, school attainment level

^d^Allergy, asthma, diabetes, epilepsy, heart condition, etc.

^e^At least once a month

**Table 9:** Associations between NSP and health behaviour variables in 14 to 16 year old Finnish adolescents

		NSP^a^				Frequent NSP^b^			
Variables	Category	Boys (*n* = 768)		Girls (*n* = 856)		Boys (*n* = 768)		Girls (*n* = 856)	
		OR^c^	95 % CI	OR^c^	95 % CI	OR^c^	95 % CI	OR^c^	95 % CI
Screen time^d^		**1.05**	**(1.00**–**1.10)**	1.03	(0.98–1.07)	1.03	(0.98–1.09)	1.02	(0.98–1.06)
Leisure time PA^e^	Approx. <30 min/week	1 (referent)		1 (referent)		1 (referent)		1 (referent)	
	Approx. 1–3 h/week	0.81	(0.40–1.65)	1.37	(0.80–2.33)	1.94	(0.53–7.11)	1.29	(0.69–2.39)
	Approx. 4–6 h/week or more	0.79	(0.37–1.66)	1.07	(0.60–1.90)	1.27	(0.31–5.15)	1.23	(0.62–2.41)
Sports club membership	No	1 (referent)		1 (referent)		1 (referent)		1 (referent)	
	Yes	1.09	(0.72–1.65)	0.76	(0.53–1.10)	0.64	(0.30–1.37)	**0.52**	**(0.33**–**0.82)**
Use of alcohol	<1 x month	1 (referent)		1 (referent)		1 (referent)		1 (referent)	
	1 x month	1.70	(0.91–3.17)	1.84	(0.97–3.49)	1.46	(0.47–4.54)	1.34	(0.68–2.62)
	≥2–3 x month	**2.75**	**(1.29**–**5.83)**	1.52	(0.77–2.99)	2.73	(0.92–8.09)	0.96	(0.45–2.06)
Smoking	No	1 (referent)		1 (referent)		1 (referent)		1 (referent)	
	Yes	1.36	(0.76–2.43)	**1.65**	**(1.04–2.59)**	1.56	(0.61–3.98)	1.42	(0.87–2.32)

Statistically significant results are indicated in bold

^a^NSP more than once a month during the last 3 months

^b^NSP at least once a week during the last 3 months

^c^Binary logistic regression was used and all variables were included in the same model. Analyses were adjusted by age, BMI, chronic diseases, smoking, school attainment level

^d^TV, computer, computer/console games, phone, tablet use, OR calculated per additional hour of screen time

^e^Intensity: breathlessness and sweating

**Table 10:** Associations between NSP and training characteristics in 14 to 16 year old sports club members

	Boys		Girls	
Training Characteristics	OR^a^	95 % CI	OR^a^	95 % CI
Active playing/practicing years (boys *n* = 488, girls *n* = 465)	0.94	(0.87–1.01)	**1.07**	**(1.00–1.14)**
Training hours per week during training season (boys *n* = 486, girls *n* = 463)	1.00	(0.96–1.04)	0.98	(0.94–1.02)
Training hours per week during competition season (boys *n* = 482, girls *n* = 448)	0.98	(0.93–1.02)	1.00	(0.96–1.04)
Number of competitions/games during previous 12 months (boys *n* = 485, girls *n* = 462)	1.00	(0.99–1.00)	1.00	(0.99–1.01)
Number of rest days during training season (boys *n* = 483, girls *n* = 461)	1.10	(0.92–1.30)	1.05	(0.91–1.21)
Number of rest days during competition season (boys *n* = 480, girls *n* = 459)	1.15	(0.96–1.37)	0.96	(0.83–1.10)

Statistically significant results are indicated in bold

^a^All training variables analysed in separate models. Adjusted by age, BMI, chronic diseases, smoking, school attainment level

Higher screen time, as calculated per additional hour of screen time (computer games, TV/DVD, phone, Internet) during leisure time, slightly increased the odds of NSP in boys, as presented in Table 9 (OR 1.05, 95 % CI 1.00–1.10). For girls, the increased odds were not statistically significant (also shown in Table 9). However, analysis also detected a significant increase in the odds for NSP among girls when screen time exceeded 4 h/day (OR 1.39, 95 % CI 1.05–1.85). Smoking increased the odds of NSP (OR 1.65, 95 % CI 1.04–2.59, Table 9) in girls. Sports club membership was associated with a lower risk for frequent NSP in girls (OR 0.52, 95 % CI 0.33–0.82). Associations were not found between NSP and training characteristics (Table 10) other than an additional year of active playing/practicing slightly increased the odds of NSP in girls (OR 1.07, 95 % CI 1.00–1.14).

## Discussion

In this multidisciplinary multicenter study, we investigated the prevalence of self-reported low back pain and neck and shoulder pain, and the related factors in members and non-members of adolescents’ sports clubs. Our findings show that self-reported low back pain (LBP) and neck and shoulder pain (NSP) are already common among adolescents. Girls seem to be at a higher risk for reporting LBP and NSP. Our results also suggest that the prevalence of LBP is higher in boys who participate in organized sports club activities. On the other hand, sports club members seem to suffer NSP less frequently than non-members do in general.

The strength of this study was the representative sample of adolescents, who were aged 14–16 years and from different regions and sizes of municipality. The sports club sample comprised the ten most popular sports in Finland. Organized sports clubs are the main setting for leisure-time physical activity in adolescents, especially in the Nordic countries. In Finland, nearly half (46 %) of children and adolescents aged 10–16 years take part in organized sports club activities [22]. Due to their wide reach and the informal educational nature, sports clubs offer a potential setting for health promotion [23]. However, even though sports clubs are positively oriented towards the idea of health promotion, the clubs’ practices have been shown to be limited and directed mainly towards sports performance and less towards other areas of health [24].

It is a common belief that those who participate in sports club activities automatically have a more physically active and healthy lifestyle than non-members. Research findings on these issues are, however, inconsistent. Three quarters of the general population of Finnish children and adolescents aged 11–15 [22, 25] and one third of sports club members in the Nordic countries do not meet the recommended level of physical activity [26–28]. In the present study, sports club members were significantly more active than non-members; nevertheless, 16 % of the members reported only approximately 1–3 h of leisure time activity per week.

We found a 49.5 % lifetime prevalence of self-reported LBP in boys, and the same prevalence for girls was 61.3 %, which is in line with the findings of Harreby at al. [29], who investigated the risk factors of LBP in a cohort of 1389 Danish children aged 12–16 years (49.8 % in boys and 67.4 % in girls) and had similar definition of LBP as our study. Van Gent et al. [30] reported 3-month prevalence of LBP in adolescents aged 12–14 years (*n* = 745) as 53.8 % for girls and 39.4 % for boys. In the present study, the prevalence of frequent LBP was in line with the results of severe LBP in the study Van Gent et al. [30] (11.0 % vs 9.5 % in girls and 5.4 % vs 4.5 % in boys, respectively). Van Gent et al. [30] defined LBP complaints severe if they bothered the children daily, demanded medication use or affected normal functioning, which is somewhat different than in our study. In our study LBP was defined as”ache or pain in the low back” and frequent LBP was reported to occur at least once a week.

Van Gent et al. [30] reported that among 12–14-years-old, the prevalence of severe NSP is 6.5 % for girls and 5.0 % for boys. Diepenmaat et al. [13] reported that among 12–16-year-olds, the prevalence of frequent NSP (more than 4 days a month) is 14.2 % for girls and 8.7 % for boys. Myrtveit et al. [31] reported that among 18-year-olds, weekly NSP was suffered by 28 % of girls and 11 % of boys. Similarly, Ståhl et al. [4] found a 19 % prevalence of weekly neck pain among of 13–16-year-old boys and girls. Our findings on the prevalence of frequent NSP are in line with these previous findings. However, the prevalence of NSP in girls was higher in our sample compared to previous studies [13, 30].

It has been suggested that the relationship between LBP and physical activity is U-shaped [32]. Some studies have found that as the intensity or amount of physical activity increases, so does the risk of LBP in the adolescents [7, 10, 12, 33]. Some studies have not been able to find an association between physical activity and LBP [13, 14, 30, 34] or the development of neck and upper limb/shoulder pain [13, 35–38]. In a recent prospective population-based cohort study among 19–21-year-old men, moderate physical activity and a good fitness level were found to protect the subjects from LBP [39]. Physical activity has been reported to be associated with a reduced risk for NSP [31]. Wedderkopp and et al. [36] did not notice significant increases in the odds of neck pain when they compared physical activity (low, mid, high) measured objectively with an accelerometer in 9-year-old children. However, they noticed that 9-year-old children with the lowest levels of physical activity were four times more likely to have low back pain 3 years later than the children with the highest levels of physical activity [36].

Mogensen et al. [34] investigated the difference of the 1-month prevalence of low back pain and neck pain in adolescents (12–13-years-old) participating in sports and those who did not take part in any sport. They found no difference between the groups for LBP (40 % vs 39 %) or neck pain (13 % vs 11 %). Even though we did not find statistically significant associations between self-reported leisure-time physical activity and LBP or NSP in the present study, we did find a significantly higher prevalence of LBP in male sports club members and a higher prevalence of NSP in non-members in general. According to a prospective study, athletes participating in sports club activities at least twice a week reported significantly more LBP than non-athletes (*n* = 116, age range 10.3–13.3) [40]. The higher prevalence of LBP in boys who are sports club members might be due to the higher volume and intensity of exercise. The increased prevalence of LBP in male sports club members might be due to insufficient recovery, as suggested by our finding on the association between LBP and the number of rest days.

In the present study, the majority of the subjects – both members and non-members – reported the origin of LBP to be overuse, and the results are in line with previous reports within athletic and general populations [11, 40–43]. In addition, previous results [4, 44] on concomitant pain being more common than single LBP or single NSP are supported by our findings.

With regard to gender, our results are in line with previous results. In general, girls are at a higher risk for developing LBP [2, 3, 9, 29] and NSP [2–4, 9, 30, 31]. However, the recent meta-analysis of LBP in children and adolescents by Calvo-Muñoz et al. [5] and the study by Schmidt et al. [45] – who studied the prevalence of LBP in adolescent athletes – found no association between gender and LBP. We found that the girls’ odds of having frequent LBP and frequent NSP were 2.33- (95 % CI 1.58–3.45) and 4.45-times (95 % CI 3.08–6.40) higher than the boys’ odds.

Interestingly our results showed a trend towards self-reported LBP being more common in non-members in girls, contrary what was seen among the boys. This might simply be a consequence of boys participating more frequently in sports with higher spinal loads (flexion and rotation), such as ice hockey and football. We found higher prevalence of frequent NSP in non-members. In relation to previous studies that have found frequent computer-related activities to increase the risk of NSP and LBP in adolescents [2] it could be speculated that the increased prevalence in the present study may be at least partly associated with the higher screen time reported by the non-members. On the other hand, NSP has also been associated with depressive symptoms and stress in a study where computer use was not found to be significantly associated with NSP or LBP [13].

It could be expected that when the amount of rest and recovery time decreases, the incidence in overuse injuries in particular increases. High frequency of training and lack of rest days are possible risk factors that sports clubs can control and thus modify the predisposing factors for injuries. In this study, no significant associations between training exposure hours per week and LBP were found in girls, which is in accordance with findings of the study by Tunas et al. [11]. However, we found a negative association between LBP and rest days and a positive association between LBP and number of competitions, and training hours in males. The number of rest days was also associated with LBP in girls, the association being negative. Schmidt et al. [45] found a statistically significant trend towards an increased prevalence of LBP in those athletes who were training the most. Ristolainen et al. [46] found that elite athletes (aged 15–35) with less than two rest days per week during the training season were more than five times more likely to report an overuse injury (95 % CI 1.89–14.06, *P* = 0.001). It is therefore important at the sports club-level to tackle the challenge of how to minimize the possibility of overload and to decrease the incidence of overuse injuries.

There are some limitations in the present study that must be acknowledged. Due to the cross-sectional design of the study, one must be cautious in drawing conclusions, especially concerning causality –that is, to differentiate the associated factors as predisposing factors or simply consequences. For example, pain may have affected training frequency or duration and influenced the physical activity or inactivity of the study subjects. In addition, there is a possible recall bias as with retrospective designs, the ability of the subject to remember and report the information correctly is a potential issue. The validity of the surveys was not studied; however, the questionnaires used in these surveys were compiled from previously validated questions in other similar studies of school-aged adolescents [16–20].

Also psychosocial factors have been shown to be associated with LBP and NSP in adolescents [44, 47]. The lacking of these variables as potential confounders could have influenced the results of this study as screen time has been shown to be associated with symptoms of depression and anxiety in adolescent [48].

## Conclusions

Self-reported low back pain and neck and shoulder pain are common among 14–16-year-olds. The prevalence of LBP was higher in male sports club members and the prevalence of NSP was higher among non-members in general. It also seems that higher screen time is weakly associated with musculoskeletal symptoms of the back, neck, and shoulder regions among adolescents.

## Abbreviations

LBP, low back pain; NSP, neck and shoulder pain

## Additional file

Additional file 1: HPSC Health behaviour survey. (PDF 197 kb) Additional file 2: HPSC MSK survey. (PDF 254 kb)
