# Supplementary material for: Low back and neck and shoulder pain in members and non-members of adolescents’ sports clubs: the Finnish Health Promoting Sports Club (FHPSC) study
Source: BMC Musculoskelet Disord. 2016 Jul 1;17:263. doi: 10.1186/s12891-016-1114-8 (PMC4930616; doi:10.1186/s12891-016-1114-8)
Supplement: Additional file 1: — HPSC Health behaviour survey. (PDF 197 kb) [file 12891_2016_1114_MOESM1_ESM.pdf]

HEALTH PROMOTING SPORTS CLUB (HPSC) STUDY FOR SPORTS CLUB MEMBERS (15y)

Hi,

This is a survey for the Health Promoting Sports Club (HPSC) study. The study is carried out in collaboration with the University of Jyväskylä, UKK-Institute and Finnish Sports Medicine Centres.

Firstly, you are asked about your opinions about actions by your coach and your group/team.. Think back and contemplate the daily actions of your group/team during the ongoing season. Secondly, the next set of questions concern your health behaviours. For this study to be applicable, it is important that you answer the questions truthfully.

Your personal details and contact information are asked in the survey. It is important to fill in these details, so that answers can be linked to the other details collected during the study. By filling in your contact details, you can also take part in a film ticket draw.

- Answering time is approximately **45** minutes.
- All information in the survey is handled confidentially.
- No one else other than approved researchers has access to the questionnaires.
- The information you'll give, will be handled unnamed and reported as larger unities. Therefore, you can answer the questions with confidence.

Thank You for your contribution! You answers are extremely important!

On behalf of the study group,

Sami Kokko
Senior researcher
University of Jyväskylä

Jari Parkkari
Chief physician
UKK-Institute,
Tampere Research Center of Sports Medicine

ID: ________

BACKGROUND INFROMATION

Name: ____

Social security number: ______________________________________

Address: ____

Phone number: ____________________________________

E-mail: ___________________________________

1. Today's Date: ____/____/ 2013

2. Gender

□ girl
□ boy

3. Year or birth (e.g. 1997) ________

4. In what month are you born?

□ January
□ February
□ March
□ April
□ May
□ June
□ July
□ August
□ September
□ October
□ November
□ December

5. Height ______cm

6. Weight ______kg

7. What kind of place do you live in?

□ In the city centre
□ City, outside city centre
□ Countryside, church village or municipal centre
□ Countryside, outside the church village or the municipal centre

8. What do you think you will do, when you finish comprehensive school?

□ Try to enter general upper secondary school
□ Try to enter vocational school/work
□ Try to get an apprenticeship
□ Double examination (e.g. general upper secondary school and vocational school)
□ Get a job
□ Be unemployed
□ I don't know

9. What was your average school grade (all subjects) in the most recent school report?

□ < 6,5
□ 6,5 - 6,9
□ 7,0 - 7,4
□ 7,5 - 7,9
□ 8,0 - 8,4
□ 8,5 - 8,9
□ 9,0 - 9,4
□ 9,5 - 10,0

10. What was your grade from your physical education lessons in the most recent school report?

□ 4
□ 5
□ 6
□ 7
□ 8
□ 9
□ 10

11. At present, how many minutes are you physically active in a week during school hours?

____ minutes (calculate a sum of the compulsory and optional school physical education, e.g., 2x45 min.=90 minutes)

12. Would you say your health is…?

□ excellent
□ good
□ fair
□ poor

13. How fit do you think you are?

□ very fit
□ fit
□ moderately fit
□ Not fit at all

14. Do you think your body is…?

□ Much too thin
□ A bit too thin
□ About the right size
□ a bit too fat
□ Much too fat

15. Do you ever feel lonely?

□ Yes , very often
□ Yes, quite often
□ Yes, sometimes
□ No

16. During the last month, have you taken any medicine or tablets for the following?

	No	Yes, once	Yes, more than once
headache	□	□	□
stomach ache	□	□	□

ID: ________

Difficulties in getting to sleep		□	□	□
nervousness	□	□	□	
ache, due to a sports injury		□	□	□
something else		□	□	□

17. During the past 12 months, how many times were you in a physical fight?

- □ I have not been in a physical fight in last 12 months
- □ 1 time
- □ 2 times
- □ 3 times
- □ 4 times or more

18. How many computers do your family own (including laptops and tablets, not including game consoles and smartphones)?

- □ None
- □ One
- □ Two
- □ More than two

19. Does your family own a car, van or truck?

- □ No
- □ Yes

20. Do you have your own bedroom for yourself?

- □ No
- □ Yes

21. How many times did you and your family travel out of [insert country here] for a holiday/vacation last year?

- □ Not at all
- □ Once
- □ Twice
- □ More than twice

22. How easy is it for you to talk to the following persons about things that really bother you? Please tick one box for each line.

	Very easy	Easy	Difficult	Very Difficult	I Don't have or see this person
Father	□	□	□	□	□
Stepfather (or mother's boyfriend)	□	□	□	□	□
Mother	□	□	□	□	□
Stepmother (or father's girlfriend)	□	□	□	□	□

PHYSICAL ACTIVITY

In the following questions physical activity is any activity that increases heart rate and makes you get out of breath some of the time. Physical activity can be done in sports, school activities, playing with friends, or walking to school. Some examples of physical activity are running, brisk walking, roller skating, cycling, dancing, skateboarding, swimming, skiing, football, basketball and Finnish baseball.

23. Over the past 7 days, on how many days were you physically active for a total of (at least) 60 minutes per day? Please add up all the time you spent in physical activity each day.

0 days 1 2 3 4 5 6 7 days

24. How many days in a regular week are you physically active for a total of (at least) 60 minutes per day?? Mark down only one option.

0 days 1 2 3 4 5 6 7 days

25. Do you think you will participate in sports or other physical activities when you are 20 years old?

□ Definitely yes
□ Probably yes
□ Probably no
□ Definitely not

26. How far is your way to school (home to school)?

□ Less than 500m
□ 500m-1 kilometre
□ 1,1-2 km
□ 2,1-3 km
□ 3,1-4 km
□ 4,1-5 km
□ more than 5 km

27. How do you travel to school during this time of the year? (mark only the most used)

□ walking
□ cycling
□ car driven by my parents
□ scooter, moped or micro car
□ other motor vehicle, like bus

28. On a daily basis, how long does it take for you to travel to school by walking, cycling or by other means that require you to be physically active? (total time for going and coming from school)

□ not at all
□ less than 20 minutes a day
□ 20–39 minutes a day
□ 40–59 minutes a day
□ hour a day or more

29. Outside school hours: how often do you usually exercise in your free time so much that you get out of breath or sweat?

□ Everyday
□ 4-6 times a week
□ 2-3 times a week
□ once a week
□ once a month
□ Less than once a month
□ Never

30. Outside school hours: how many hours per week do you usually exercise in your free time so much that you get out of breath or sweat?

□ None
□ About.half an hour

□ About 1 hour
□ About 2-3 hours
□ About 4-6 hours
□ About 7 hours or more

31. <u>Outside school hours</u>: how often do you do the following sports in your free time? <u>Choose the option that best describes the average situation during the last year (during the time of the year that it is appropriate to do the specific sport).</u>

	Never	Once a month or less	2-3 times a month	Once a week	2-3 times a week	4 times a week or more often
• walking	□	□	□	□	□	□
• running, jogging						
• cycling						
• football						
• floorball						
• basketball						
• Finnish baseball						
• volleyball						
• badminton						
• tennis						
• table tennis						
• gym, weight training						
• swimming						
• other aquatic activities (e.g. diving, water polo, kayaking)						
• cross-country skiing						
• skiing, snowboarding						
• games on ice (e.g. ice hockey, ringette, bandy)						
• figure skating						
• gymnastics, fitness (e.g. aerobic, cheerleading, zumba)						
• dance						
• yoga/Pilates						
• riding						
• track and field						
• roller skating						
• skateboarding						
• martial arts (e.g. judo, taekwondo, karate, wrestling, boxing)						
• golf						
• orienteering						
• bowling						
• outdoor games and -play (e.g. parkour, sledging, tobogganing, Frisbee golf)						
• motor sports						
• something else, what?						

32. In a typical week: How often does your mother (or step- mother, if your mother does not live in your primary home))…

	Never	Rarely	Sometimes	Often	Very often	□ I don't have or don't meet her
Encourages you to be physically active or to play sports	□	□	□	□	□	
Gives you a ride to a place where you can be physically active/exercise or						

the place of your sport	□	□	□	□	□
Is physically active/exercises or plays sports with you	□	□	□	□	□

33. On a typical week: How often does your father (or step-father, if your father does not live in your primary home))…

	Never	Rarely	Sometimes	Often	Very often	□ I don't have or don't meet him
Encourages you to be physically active or to play sports	□	□	□	□	□	
Gives you a ride to a place where you can be physically active/exercise or the place of your sport	□	□	□	□	□	
Is physically active/exercises or plays sports with you	□	□	□	□	□	

34. here is a list of the reasons that some young people give for taking part in physical activity in their free time. For each reason please tick how important it is for you. Please tick one box for each line.

I exercise//I'm physically active because…	Very important	Fairly important	Not important
to have fun	□	□	□
to be good in sport	□	□	□
to win	□	□	□
to make new friends	□	□	□
to improve my health	□	□	□
to see my friends	□	□	□
to get in good shape	□	□	□
to look good	□	□	□
I enjoy the feeling of using my body	□	□	□
to please my parents	□	□	□
to be cool	□	□	□
to control my weight	□	□	□
it is exciting	□	□	□
to lose weight	□	□	□
to gain muscle mass	□	□	□

SPORTS

The following questions concern mainly the hobby you do within your sports club. Choose the option that best describes you or your team/group. Mark your answer with an X or by writing your answer over the line.

35. What is your primary sport (the sport that you do the most)?___________________

36a. Do you take part in other sports (within a sports club)?

□ Yes, my 2nd sport is:_____________, my 3th sport is:_____________,
my 4th sport is :______________ □ No

36 b. If you answered no, would you be willing to take up another sport (within a sports club)?

□ Yes
□ No

36 c. If you answered yes, what is the main reason for not having another sport hobby within a sport club at present?

□ I just have not figured it out yet
□ Because of my primary sport, I don't have time
□ My coach has told me not to take part in another sport
□ It would b too expensive and my family can't afford it
□ Some other reason, what?_______________________________________

37. What is the name of the sports club you do your primary sport within? (select from below)

□ Jyväskylä xxx sports club…
□ Tampere xxx…
□ etc.

38. At what age did you start your primary sport (e.g. 12-years-old)?

_____ years old

The following questions concern your primary sport, unless otherwise mentioned.

39. What is the level of the league or games you take part in?

□ National
□ Other than national, such as 1th division
□ Local/district
□ Neighbourhood/city
□ No/we don't take part in games/competitions

40. On a typical week, how many times do you have…

a) coach-led training ____times (none = 0 times)
b) games/competitions ____times
c) self-directed training ____times

41. What is the duration of a typical training?

a) coach-lead training ____ minutes (1 hour = 60 minutes)
b) self-directed training ____ minutes

42. In your spare time, in addition to your primary sport: how often are you physically active in a week? (at least ½ an hour so that your sweat)

a) other sports ____times (none = 0 times)
b) self-directed training e.g. playing with friends (other than self-directed training of the primary sport). ____times

43. How often do you perform warm up and cool down?

Warm up

a) organized (coach-led)

□ every time
□ almost always
□ every now and then
□ never

b) self-directed
□ every time
□ almost always
□ every now and then
□ never

Cool down

a) organized (coach-led)
□ every time
□ almost always
□ every now and then
□ never

b) self-directed
□ every time
□ almost always
□ every now and then
□ never

44. What are your competitive goals as an athlete? Select your highest goal.

□ I don't have competitive goals, doing sports is a hobby to me
□ I don't have competitive goals, I play sports to improve physical fitness
□ Competitive success at junior local or district level/□ Competitive success at junior national level
□ Competitive success at junior international level
□ Competitive success as an adult at national level competitions
□ Competitive success as an adult at the international level (European, world championships or professional athlete)

45a. Have you ever had a conversation with your coach concerning your long term competitive goals?

□ Yes
□ No

45b. If you answered yes, did your coach tell you what it takes regarding a) training and b) life outside training?

a) training
☐ yes
☐ no

b) **life outside training**
c) ☐ yes
d) ☐ no

45c. If you answered no, would you have liked for your coach to discuss your goals as an athlete with you??

☐ Yes
☐ No

46. Do you train enough with relation to your goals?

☐ Yes
☐ No

47. How far is the journey from your home to the main training location of your primary sport? Accuracy of 1 kilometre

□ less than 500m
□ 500m-1 kilometre
□ 1,1-2 km
□ 2,1-3 km
□ 3,1-4 km
□ 4,1-5 km
□ 5,1-6 km
□ 6,1-7 km
□ 7,1-8 km
□ more than 8 km

48. At this time of the year, how do you mainly travel the journey from home to the training location? (choose one, the most frequently used mode of travel)

□ walking
□ cycling
□ by car with my parents
□ scooter, moped or micro car
□ other motor vehicle

49. How much time does the travel form home to the location of your training usually take when you walk, cycle or use other ways of travelling that demands physical activity? (trip there and back in total)

□ I do not travel that demands physical activity
□ less than 20 minutes a day
□ 20–39 minutes a day
□ 40–59 minutes a day
□ one hour a day or more

50. Over the last 12 months, how many times have you come across violence or threat of violence in sports?

□ not at all
□ 1 time
□ 2 times
□ 3 times
□ 4 times or more

51. Do you have the possibility to visit a sports psychologist through your sports club?

□ Yes
□ No
□ I don't know

52. Over the last 12 months, how many times have you had a discussion with a sports psychologist about matters concerning your sport participation?

□ not at all
□ 1 time
□ 2 times
□ 3 times
□ 4 times or more

ID: ________

HEALTH PROMOTION ACTIVITY OF THE COACHES

53. How many coaches are there in your group/team?

□ 1
□ 2
□ 3
□ 4 or more

54. How long has the head coach been your/your teams' coach?

□ less than a year
□ 1-2 years
□ 3-4 years
□ 5 years or longer

55. In your opinion, over the last 6 months, how often has your head coach taken into account the following matters during training/games?

	never	rarely	often	very often
a) coach-led training load is too heavy				
b) coach underlines that training should be fun				
c) coach has told us not to chat during training				
e) coach makes sure that training is safe				
d) training takes place at a suitable time of the day				
f) the whole group/team trains in a same way				
g) the best in our group/team get more attention from our coach				
i) coach underlines, that other athletes should be respected				
h) coach give negative feedback every time someone make a mistake/fails				
j) coach always acts, if someone in our group/team breaks the rules of our sport				

56. In your opinion, over the last 6 months, how often has your head coach taken into account the following matters during non-performance time within club activities, i.e. time other than practice/competition, such as locker room or excursion activities?

	never	rarely	often	really often
a) There is a balance between trainings/games and rest days on weekly basis				
b) Coach instructs what to eat while eating together				
c) Coach motivates us to be socially interactive with the team/group members outside practice/competition				
d) Exertion caused by school work is in balance with relation to training; i.e., individual's total weekly exertion is in balance				
e) coach discuss on health issues also with parents				
f) coach recognises matters outside sports with relation to coaching				
g) coach always intervene in possible use of snuff or smoking				
i) coach have given us a lecture or separate info on some health topic				
j) coach has given information leaflets on some health topic				
k) outside expert has been talking to us about health issues				
l) coach has talked to us about risks of substance use				

57. In your opinion, over the last 6 months, how often has your head coach discussed at least basic issues on the following health topics with your team/group (in individual sports with you)?

	never	rarely	often	really often
a) physically active lifestyle				
b) nutrition				
c) smoking				
e) alcohol				

d) snuff
f) drugs
g) doping
h) sleep/rest
j) injury prevention
i) hygiene
k) risks of training while ill/sick
l) violence that relates to sport in question
m) sexual issues

HEALTH BEHAVIOUR AND OTHER HOBBIES

58. How many hours a day do you sit during a regular day at school? Sum up all time spent sitting down such as lessons, at school, breaks, and other time spent in school. Write down the hours, with ½ an hour accuracy.

___hours

59. How many hours a day do you spend sitting down in front of a television, computer/tablet, phone or other similar device? Write down the hours, with ½ an hour accuracy to each device separately for school day and weekend.

SCHOOLDAY

Morning before school

____ hours TV, videos or DVD
____ hours computer or console games (including phone and tablet games)
____ hours computer/tablet (other than playing games, for example chatting, internet, e-mail, homework)

Afternoon and evening after school

____ hours TV, videos or DVD
____ hours computer or console games (including phone and tablet games)
____ hours computer/tablet (other than playing games, for example chatting, internet, e-mail, homework)

Estimate how many hours do you spend sitting on before going to school and after school on a regular school day

_____ hours

WEEKEND

Morning and afternoon (6AM-6PM)

____ hours TV, videos or DVD
____ hours computer or console games (including phone and tablet games)
____ hours computer/tablet (other than playing games, for example chatting, internet, e-mail, homework)

Evening (6PM-Midnight)

____ hours TV, videos or DVD
____ hours computer or console games (including phone and tablet games)
____ hours computer/tablet (other than playing games, for example chatting, internet, e-mail, homework)

Late evening/night (Midnight-6AM)

____ hours TV, videos or DVD

____ hours computer or console games (including phone and tablet games)
____ hours computer/tablet (other than playing games, for example chatting, internet, e-mail, homework)

Estimate how many hours do you spend sitting on a regular weekend day
____ hours

60. How often do you usually have breakfast (more than a glass of milk or fruit juice)? Please tick one option for weekdays and one for weekend

Schooldays
□ I never eat breakfast during the week
□ one day
□ two days
□ three days
□ four days
□ five days

Weekend
□ I never have breakfast during the weekend
□ I usually have breakfast on only one day of the weekend (Saturday OR Sunday)
□ I usually have breakfast on both weekend days (Saturday AND Sunday)

61. How often do you skip school lunch in a regular school week?
□ Not once; I usually always eat lunch at school
□ once a week
□ twice a week
□ three days a week
□ four days a week
□ five days a week

62. How often do you usually drink or eat the following things? Mark down one option fro each food/drink.

	Never	Less than once a week	Once a week	2-4 days a week	5-6 days a week	Every day, once a day	Every day, more than once
salad							
fruit							
vegetables							
candy (candy or chocolate)							
soft-drinks or other soda, that contain sugar							
dark bread (rye bread, rye crisp etc.)							
hamburgers or hot dogs							
potato or corn chips							
energy drinks (such as Battery, Red Bull)							
sports drinks							
non-fat milk, low fat milk or semi-skimmed milk							
full fat milk							
pizza							
vitamins							
other dietary supplements (such as protein, maltodextrin, creatine or similar)							

ID: ________

63. How often do you brush your teeth?

□ More than once a day
□ Once a day
□ At least once a week, but not daily
□ Less than once a week
□ Never

64. Have you experienced toothache or some other mouth related complaint over the last 12 months ?

□ Yes
□ No

65. When do you usually go to bed… (select the right time)

a) **If you have to go to school the next morning? (but no training or games/competition)?**

At the latest 21.00	21.30	22.00	22.30	23.00	23.30	24.00	00.30	01.00	01.30	02.00 or later

b) **If you have to go to school the next morning (and there is a training or games/competition where you live; no travelling needed)?**

At the latest 21.00	21.30	22.00	22.30	23.00	23.30	24.00	00.30	01.00	01.30	02.00 or later

c) **on Friday or Saturday evenings, when you have an early morning start the next day due to training, game or competition?**

At the latest 21.00	21.30	22.00	22.30	23.00	23.30	24.00	00.30	01.00	01.30	02.00 or later

d) **On Friday or Saturday evenings, when you are free from training the next day?**

At the latest 21.00	21.30	22.00	22.30	23.00	23.30	24.00	00.30	01.00	01.30	02.00 or later

66. When do you usually go to bed…

a) **On Friday or Saturday evenings, when you have had training or game/competition?**

At the latest 21.00	21.30	22.00	22.30	23.00	23.30	24.00	00.30	01.00	01.30	02.00 or later

b) **On Friday or Saturday evenings, when you are free from training the next day?**

At the latest 21.00	21.30	22.00	22.30	23.00	23.30	24.00	00.30	01.00	01.30	02.00 or later

67. When do you usually wake up on school mornings? ?

no later than 05.00	05.30	06.00	06.30	07.00	07.30	08.00 or later

68. At what time do you usually wake up …

a) on a school day after coming home late, due to late training session or game/competition

no later than 05.00	05.30	06.00	06.30	07.00	07.30	08.00	08.30	09.00	09.30	10.00

b) Saturday or Sunday mornings, when I have training or game/competition

no later than 05.00	05.30	06.00	06.30	07.00	07.30	08.00	08.30	09.00	09.30	10.00

10.30	11.00	11.30	12.00	12.30	13.00	13.30	14.00 or later

c) Saturday or Sunday mornings, when I don't have training or game/competition

no later than 05.00	05.30	06.00	06.30	07.00	07.30	08.00	08.30	09.00	09.30	10.00

10.30	11.00	11.30	12.00	12.30	13.00	13.30	14.00 or later

69. Have you ever smoked tobacco? (at least one tobacco, cigar or tobacco pipe)

□ Yes □ No

70. How often do you smoke tobacco at present?

□ Every day
□ At least once a week, but not every day
□ Less than once a week
□ I do not smoke

71. Have you ever tried snuff?

□ No I haven't tried
□ I have tried once or twice
□ I have tried more than twice

72. Do you use presently use snuff?

□ Yes very day
□ Yes every week, but not dailyz
□ Yes, but less than once a week
□ I don't use snuff

73. Have you ever tried alcohol?

□ No
□ Yes I have tried

74. In total, how often do you use alcohol, for example half a bottle of medium strength beer or more?

□ Once a week or more often
□ Few times a month
□ Approx. once a month
□ Less than once a month
□ I don't use alcohol

75. Have you ever had so much alcohol, that you have been really drunk?

□ No never
□ Yes, once
□ Yes, 2-3 times
□ Yes, 4-10 times
□ Yes, more than 10 times

76. <u>Outside of school hours</u>: How often do you do the following things? Select the option that describes the situation for you during the last 12 months (at the time it is possible to do the mentioned activity).

	Never	Once a month or less	2-3 x a month	Once a week	2-3 x a week	4 x a week or more
• Watching TV/DVD	□	□	□	□	□	□
• Using computer	□	□	□	□	□	□
• Spending time with friends	□	□	□	□	□	□
• Taking care of pets	□	□	□	□	□	□
• Listening to music	□	□	□	□	□	□
• Playing music or singing	□	□	□	□	□	□
• the scouts/4H/etc.	□	□	□	□	□	□
• crafts/building/handiworks	□	□	□	□	□	□
• reading	□	□	□	□	□	□
• going to movies/theatre/ concerts	□	□	□	□	□	□
• cooking	□	□	□	□	□	□
• disco/going out dancing	□	□	□	□	□	□
• art (e.g. painting/drawing)	□	□	□	□	□	□
• camping/hunting/fishing	□	□	□	□	□	□
• photography	□	□	□	□	□	□
• sports spectating competitions/games at the site	□	□	□	□	□	□
• riding a moped	□	□	□	□	□	□
• Games (e.g. board games, sudoku, crosswords)	□	□	□	□	□	□
• something else, what?	□	□	□	□	□	□

THANK YOU FOR YOUR ANSWERS!!!
