# Supplementary material for: Low back and neck and shoulder pain in members and non-members of adolescents’ sports clubs: the Finnish Health Promoting Sports Club (FHPSC) study
Source: BMC Musculoskelet Disord. 2016 Jul 1;17:263. doi: 10.1186/s12891-016-1114-8 (PMC4930616; doi:10.1186/s12891-016-1114-8)
Supplement: Additional file 2: — HPSC MSK survey. (PDF 254 kb) [file 12891_2016_1114_MOESM2_ESM.pdf]

**9th graders: MUSCULOSKELETAL HEALTH SURVEY**

With this survey, information concerning the musculoskeletal symptoms, back pain as well as accidents and injuries reported by the 9th graders are gathered. Read the questions carefully and choose the most appropriate option or write your answer to the space reserved for it.

Your answers are equally as important even if you haven't had back problems or haven't had accidents or injuries.

## A1. BACKGROUND INFORMATION

Name: ___________________________________________

Social security number: _____________________________________

Address: _____________________________________________________________________________

Phone number: ___________________________________

E-mail: ___________________________________

**2. Gender**

1. girl
2. boy

**3. Height ______cm**

**4. Weight ______kg**

**5. Do you have a chronic disease?**

1. No
2. Allergy
3. Asthma
4. Diabetes
5. Epilepsy
6. Heart condition
7. Other, what? ______________________________

**6. Do you have regular medication? E.g. allergy medication in the spring time.**

1. No
2. Contraceptives or other hormonal preparation
3. Allergy medication
4. Asthma medication
5. Insulin
6. Epilepsy medication

7. Heart or blood pressure medication
8. Other, what? ___________________________

**7. Over the last month, have you used NSAID (nonsteroidal anti-inflammatory drugs) or pain killers? (e.g. Burana, Ibusal, Panadol, Pamol, Voltaren)**

- ☐ **No**
- ☐ **Yes**

*(If you answered yes)* **7b Why did you take NSAIDs?** (e.g. headache, groin pain)
**What was the dose?** (e.g. Burana 400mgx3)
**Who advised you to take it?** (e.g. myself, coach, doctor, dad)

**8. Do you follow a special diet?**

- ☐ No, I follow a regular diet
- ☐ Yes, vegetarian
- ☐ Yes, Low-carb
- ☐ Yes, lactose free
- ☐ Yes, dairy free
- ☐ Yes, gluten free
- ☐ Yes, other, What? _____________________________________________________

*(if you answered yes)* **8b. Why do you have a special diet?** (e.g. allergy)

_____________________________________________________________________________

_____________________________________________________________________________

**9. Do you use dietary supplements?** (e.g. multivitamin, D-vitamin, Calcium, Omega-3, protein supplements, recovery drinks, amino acid products, creatine)

- ☐ No
- ☐ Yes, what products?

_____________________________________________________________________________

_____________________________________________________________________________

**10. Do you smoke?**

- ☐ No
- ☐ I have stopped smoking or I try to stop (not smoking currently)
- ☐ I smoke less than once a week
- ☐ I smoke once a week or more, but not daily
- ☐ I smoke daily, at least once

**The following four questions are directed for girls (**if you are a boy, there is no need to answer)

**11. Have you had your menarche?**

- ☐ No
- ☐ Yes, at what age? (in numbers) _________________

**12. Typically, how long is your menstrual cycle?** (Count it from the beginning of the most recent periods to the last day before the next periods) write the answer in numbers________________

**13. How many periods have you had over the last 12 months?** Write the answer in numbers

**14. Have your periods been heavier than usual?**

- ☐ No
- ☐ Yes

**A2. SPORT AND TRAINING INFORMATION**

**15. Are you involved in sports club activities?**

1. No (move to the question number 28)
2. Yes

In this section, questions related to training and competing over the last 12 months are asked. Even if some questions are difficult, for example the training volume, please do not skip the question, but try to make as precise estimation as possible. If you have transferred to a lower level or changed sport, answer the questions in relation to the sport you were training for at the beginning of the season before the change.

**16a. Your primary sport:** ____________________________________________________________

**16b. If your sport is a team sport, what is the main position you play in the field?**

1. My sport is not a team sport
2. Goalkeeper
3. Defence
4. Offence
5. Other, what? ____________________________________________________

**17. How old were you when you started?** Write the answer in numbers ___________________________

**18. How many years you have trained actively (at least 2 times a week)?** Write the answer in numbers ____________

**19. Do you also take part in other sports? (**We mean taking part in other sports as sports that are not part of the training of your primary sport)

1. No
2. Yes, What? ____________________________________________________________

**20. On average, how many times a week do you train (all sports in total) during the training season?**
For example, in ice hockey training season is typically from May to August. Training during competitive season is asked in another question. Write the answer in numbers, training times/week ___________

**On average, how many hours a week do you train (all sports in total) during the training season?**
Training during competitive season is asked in another question. Write the answer in numbers, training hours/week ___________

**21. On average, how many times a week do you train (all sports in total) during the competitive season?**
For example, in ice hockey competitive season is from September to April. Training during training season was already asked. Write the answer in numbers, training times/week ___________

**On average, how many hours a week do you train (all sports in total) during the competitive season?**

Training during competitive season is asked in another question. Write the answer in numbers, training hours/week ____________

**22. How many competitions did you attend or how many games did you have over the last 12 months?** Write the answer in numbers_____________

**23. On average, how many rest days (when you don't have any planned training) do you have in a week during the training season?** Write the answer in numbers _____________

**24. On average, how many rest days (when you don't have any planned training) do you have in a week during the competitive season?** Write the answer in numbers _____________

## A3. ACCESORY WORK

In this section information related to accessory work is investigated. In this case, by accessory work we mean warm up, cool down and stretching.

**25. Do you do warm up exercises, before training or competition?**

1. No
2. Sometimes
3. Yes

**26. Do you do cool down exercises, after training or competition?**

1. No
2. Sometimes
3. Yes

**27. How often do you do stretching?**

1. Not at all
2. Less than once a week
3. 1-3 times a week
4. Daily or almost daily

## B. SYMPTOMS AND INJURIES

Questions concerning your symptoms and injuries consist of four sections: 1) symptoms, 2) traumatic injuries, 3) overuse injuries and 4) low back pain. Follow the instruction in each section.

## B1. SYMPTOMS

**28. Over the last 3 months, how often have you had the following symptoms?**
(Body parts A-I in the accompanying picture)

| | Almost daily | More than once a week | Approximately once a week | 2-3 times a month | Approximately once a month | Less frequently or never |
|---|---|---|---|---|---|---|
| Headache (A) | ☐ | ☐ | ☐ | ☐ | ☐ | ☐ |
| Neck and shoulder pain or ache (B) | ☐ | ☐ | ☐ | ☐ | ☐ | ☐ |

| | | | | | | |
|---|---|---|---|---|---|---|
| Upper extremity pain or ache (C) | ☐ | ☐ | ☐ | ☐ | ☐ | ☐ |
| Thoracic pain or ache (D) | ☐ | ☐ | ☐ | ☐ | ☐ | ☐ |
| Upper back pain or ache (E) | ☐ | ☐ | ☐ | ☐ | ☐ | ☐ |
| Low back pain or ache (F) | ☐ | ☐ | ☐ | ☐ | ☐ | ☐ |
| Stomach ache (G) | ☐ | ☐ | ☐ | ☐ | ☐ | ☐ |
| Groin pain (H) | ☐ | ☐ | ☐ | ☐ | ☐ | ☐ |
| Buttock pain or ache (I) | ☐ | ☐ | ☐ | ☐ | ☐ | ☐ |
| Lower extremity pain or ache (J) | ☐ | ☐ | ☐ | ☐ | ☐ | ☐ |
| Difficulty of getting to sleep | ☐ | ☐ | ☐ | ☐ | ☐ | ☐ |
| Waking up during night time | ☐ | ☐ | ☐ | ☐ | ☐ | ☐ |

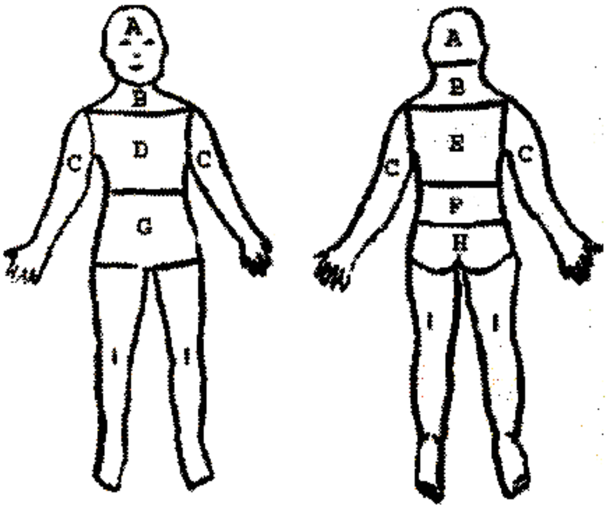


**29. Over the last 12 months, have you had an injury to any of the previously mentioned body parts?** (e.g. falling or tripping, sports injury etc.)

1. No
2. Yes, What part? (A-I) ____________________

**B2. TRAUMATIC INJURIES**

In this section, information related to traumatic injuries is investigated. Carefully read the following definition before continuing to answer the questions.

**Traumatic injury** happens suddenly or accidentally and results in discontinuing the training or sports performance. Overuse injuries are investigated separately in the section B3.

**30. Over the last 12 months, have you had a traumatic injury or an accident?**

1. No (move to question 39)
2. Yes

Choose maximum of three (3) traumatic injuries you have had over the last 12 months. Choose an option that describes the injury the most. Numbering in the questions stays the same. If you have had more than 3 injuries over the last 12 months, mark only the 3 most severe ones.

The most severe injury (injury 1) is the injury that resulted in longest time off from school, hobby/sport or competition/game or resulted in a permanent disability that resulted in you to stop playing sports. The second most severe injury is marked under injury 2 and 3th severe injury is marked under injury 3.

**31. In which body part did the injury occur?**

| | Injury 1 | Injury 2 | Injury 3 |
|---|---|---|---|
| 1. Toes | | | |
| 2. Instep of the foot | | | |
| 3. Sole of the foot | | | |
| 4. Heel | | | |
| 5. Achilles | | | |
| 6. Ankle | | | |
| 7. Shin | | | |
| 8. Calf | | | |
| 9. Knee | | | |
| 10. Back of the thigh | | | |
| 11. Front of the thigh | | | |
| 12. Hip | | | |
| 13. Buttocks | | | |
| 14. Pelvis | | | |
| 15. Groin | | | |
| 16. Stomach | | | |
| 17. Lower back | | | |
| 18. Chest | | | |
| 19. Upper back | | | |
| 20. Neck | | | |
| 21. Face | | | |
| 22. Teeth | | | |
| 23. Eye area | | | |
| 24. Head, other area | | | |
| 25. Collar bone | | | |
| 26. Shoulder area | | | |
| 27. Shoulder joint | | | |
| 28. Upper arm | | | |
| 29. Elbow | | | |
| 30. Forearm | | | |
| 31. Wrist | | | |
| 32. Palm | | | |
| 33. Thump | | | |
| 34. Other fingers | | | |
| 35. Mental injury | | | |

**32. What type of traumatic injury was it? Mark injury 1 as the injury that was injury 1 also in the previous question and so on.**

| | Injury 1 | Injury 2 | Injury 3 |
|---|---|---|---|
| 1. Concussion | | | |
| 2. Sprain, strain (e.g. knee, ankle, shoulder, finger) | | | |
| 3. Muscle cramp or tear (e.g. back, thigh) | | | |
| 4. Joint subluxation | | | |
| 5. Blow injury, contusion or bruise to face or head | | | |
| 6. Blow injury, contusion or bruise to other part of the body | | | |
| 7. Eye injury | | | |
| 8. Tooth injury | | | |
| 9. Wound | | | |
| 10. Fracture | | | |
| 11. Mental injury | | | |
| 12. Other injury | | | |

**33. In what kind of activity or sport did the traumatic injury occur?** Write the sport/activity to the corresponding columns with the injuries you have reported in the previous questions.

| | Injury 1 | Injury 2 | Injury 3 |
|---|---|---|---|
| Sport/activity where the injury 1 occurred. (For example: Trampoline jumping) | X | | |
| Sport/activity where the injury 2 occurred. (For example: Floorball) | | X | |
| Sport/activity where the injury 3 occurred. (For example: Walking) | | | X |

**34. What was the situation the traumatic injury occurred in?**

| | Injury 1 | Injury 2 | Injury 3 |
|---|---|---|---|
| 1. Tackling | | | |
| 2. Kick from a fellow player | | | |
| 3. Coalition | | | |
| 4. Falling, tripping | | | |
| 5. Slipping | | | |
| 6. Running | | | |
| 7. Stepping to the side/ turning | | | |

| | | | |
|---|---|---|---|
| 8. Slowing down/changing direction | | | |
| 9. Take off | | | |
| 10. Landing | | | |
| 11. Pass/throw | | | |
| 12. Shot | | | |
| 13. Dropping/falling | | | |
| 14. Technical error | | | |
| 15. Excessive stress | | | |
| 16. Someone hitting with a hand or fist | | | |
| 17. Someone hitting with a stick or similar | | | |
| 18. Blocking | | | |
| 19. Reaching or a playing equipment | | | |
| 20. Stretching | | | |

**35. Where did the injury occur?**

| | Injury 1 | Injury 2 | Injury 3 |
|---|---|---|---|
| 1. During my spare time hobby, not in competitive sport (e.g. in skateboarding, trampoline, jogging, gym, riding, walking snowboarding, skiing) | | | |
| 2. On my way to/from school | | | |
| 3. Physical activity lessons at school | | | |
| 4. During break/recess at school | | | |
| 5. Some other time during school hours | | | |
| 6. Competitive sport: Self-directed training | | | |
| 7. Competitive sport: coach-directed training | | | |
| 8. On my way to the training | | | |
| 9. During game/competition | | | |
| 10. During warm up/cool down | | | |

**36. Try to estimate how long of a break did you have to take from training, due to the traumatic injuries in total over the last 12 months?**

All injuries in total ___________months ___________weeks ____________days

**37. How was the traumatic injury treated?**
**Note: In this question you can choose several options.** Immediate first aid means ice, compression or elevated position of the injured body part, performed by yourself or by someone else.

| | Injury 1 | Injury 2 | Injury 3 |
|---|---|---|---|
| 1. I didn’t receive treatment | | | |
| 2. Treatment independently | | | |
| 3. Immediate first aid | | | |
| 4. NSAID e.g. Burana, Panadol | | | |
| 5. Other medication | | | |
| 6. I visited a public health nurse | | | |
| 7. I visited a doctor | | | |

| | | | |
|---|---|---|---|
| 8. Support/taping or other aid equipment | | | |
| 9. Physical therapy | | | |
| 10. I was checked in to a hospital ward | | | |
| 11. I got a plaster cast or a splint | | | |
| 12. I needed a surgery | | | |

**38. Over the last 12 months, have you had any accidents on your way to or from school or hobbies?**
(Not during school hours or during the hobby itself)

1. No
2. Yes, How many? _______________

How long was the break from your hobby, or sport due to the accident? I had to take a break from my hobby/sport for ____months _______ days

Please tell us in more detail, what type of accident was it? (E.g. fell over while riding a bike to school)

__________________________________________________________________________________________
__________________________________________________________________________________________

**B3. OVERUSE INJURIES**

In this section information related to overuse injuries is investigated. Carefully read the following definition before continuing to answer the questions.

**Overuse injury** is an injury that results in pain during stress without any observable injury. Overuse injury becomes worse, progressively and results in pain that is felt during or after exertion. Pain gets worse when continuing the stress and may result in you having to stop the performance or playing the sport.

**39. Over the last 12 months, have you had an overuse injury?**
1. No (Continue to the question number 48)
2. Yes, How many (separate) overuse injuries? ____________

Choose maximum 3 overuse injuries you have had over the last 12 months. Choose an option that describes the injury the most. Numbering in the questions stays the same. If you have had more than 3 injuries over the last 12 months, mark only the 3 most severe ones.

The most severe injury (injury 1) is the injury that resulted in longest time off from school, hobby/sport or competition/game or resulted in a permanent disability that resulted in you to stop playing the sport. The second most severe injury is marked under injury 2 and 3th severe injury is marked under injury 3.

**40. In which body part did the injury occur?**

| | **Injury 1** | **Injury 2** | **Injury 3** |
|---|---|---|---|
| 1. Toes | | | |
| 2. Instep of the foot | | | |

| | | | |
|---|---|---|---|
| 3. Sole of the foot | | | |
| 4. Heel | | | |
| 5. Achilles | | | |
| 6. Ankle | | | |
| 7. Shin | | | |
| 8. Calf | | | |
| 9. Knee | | | |
| 10. Back of the thigh | | | |
| 11. Front of the thigh | | | |
| 12. Hip | | | |
| 13. Buttocks | | | |
| 14. Pelvis | | | |
| 15. Groin | | | |
| 16. Stomach | | | |
| 17. Lower back | | | |
| 18. Chest | | | |
| 19. Upper back | | | |
| 20. Neck | | | |
| 21. Face | | | |
| 22. Teeth | | | |
| 23. Eye area | | | |
| 24. Head, other area | | | |
| 25. Collar bone | | | |
| 26. Shoulder area | | | |
| 27. Shoulder joint | | | |
| 28. Upper arm | | | |
| 29. Elbow | | | |
| 30. Forearm | | | |
| 31. Wrist | | | |
| 32. Palm | | | |
| 33. Thump | | | |
| 34. Other fingers | | | |
| 35. Mental injury | | | |

**41. Which tissue was affected?**

| | Injury 1 | Injury 2 | Injury 3 |
|---|---|---|---|
| 1. Muscle | | | |
| 2. Tendon | | | |
| 3. Bone | | | |
| 4. Joint | | | |
| 5. Ligament | | | |
| 6. Nerve tissue | | | |

**42. What kind of overuse injury was it?**

| | Injury 1 | Injury 2 | Injury 3 |
|---|---|---|---|
| 1. Pain in the muscle | | | |

| | | | |
|---|---|---|---|
| after physical activity | | | |
| 2. Overuse pain in heel | | | |
| 3. Overuse pain in knee | | | |
| 4. Overuse pain in buttock | | | |
| 5. Overuse pain in groin | | | |
| 6. Overuse pain in upper arm | | | |
| 7. Overuse pain in back | | | |
| 8. Pain in tendon | | | |
| 9. Bursitis | | | |
| 10. Nerve impingement | | | |
| 11. Stress facture | | | |
| 12. Other, what? | | | |

**43. In what kind of activity or sport did the overuse injury present itself?** Write the sport/activity to the corresponding columns with the injuries you have reported in the previous questions.

| | Injury 1 | Injury 2 | Injury 3 |
|---|---|---|---|
| Sport/activity where the injury 1 occurred. (For example: running) | X | | |
| Sport/activity where the injury 2 occurred. (For example: Floorball) | | X | |
| Sport/activity where the injury 3 occurred. (For example: Swimming) | | | X |

**44. Try to estimate how long of a break did you have to take from training, due to the overuse injuries in total over the last 12 months?**

All injuries in total ___________months ___________weeks ____________days

**45. How was the overuse injury treated?**
**Note: In this question you can choose several options.**

| | Injury 1 | Injury 2 | Injury 3 |
|---|---|---|---|
| 13. I didn’t receive treatment | | | |
| 14. Treatment independently | | | |
| 15. Ice, elevated position and compression | | | |
| 16. NSAID e.g. Burana, Panadol | | | |
| 17. Other medication | | | |

| | | | |
|---|---|---|---|
| 18. I visited a public health nurse | | | |
| 19. I visited a doctor | | | |
| 20. Support/taping or other aid equipment | | | |
| 21. Physical therapy | | | |
| 22. I was checked in to a hospital ward | | | |
| 23. I got a plaster cast or a splint | | | |
| 24. I needed a surgery | | | |

**46. Do you feel that the overuse injury occurred partly due to environmental factors**? (e.g. floor material, location of sports performance, weather?)

1. No
2. Yes, what kind of environmental factors?

   ________________________________________

   ________________________________________

**47. Do you feel that your shoes were involved with the occurrence of the overuse injury?**

1. No
2. Yes, what kind of shoes?

   ________________________________________

   ________________________________________

**48. In school, have you been informed about the prevention of leisure-time traumatic and overuse injuries?**

1. No
2. Yes

**49. In the sport club, have you been informed about the prevention of leisure-time traumatic and overuse injuries?**

1. No/ I don’t participate in organized sport club activities
2. Yes

**50. Have you been doing exercises with the purpose for preventing injuries in your sports club?**

1. No/ I don’t participate in organized sport club activities
2. Yes

**B4: LOW BACK PAIN**

This survey continues for few more questions that investigate the function and possible problems of your low back.

Low back pain is ache or pain in your lower back and pain referring to your buttocks, lower extremity (one or both) might also be present.

Choose the option that describes your situation the best.

**51. Have you ever experienced problems in your lower back?**

(area illustrated by a picture) (pain, ache or feeling of discomfort etc.)

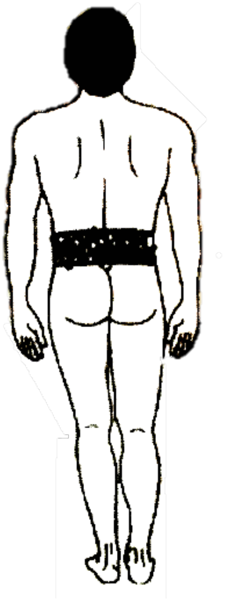

-  No (move to the last question)
-  Yes

**52. Have you had low back pain during the previous seven days?**

-  No
-  Yes

**53. In total, how many days have you had low back pain over the last 12 months?**

-  0 days
-  1-7 days
-  8-30 days
-  More than 30 days, but not daily
-  Daily

**54. Have you experienced low back pain that has required consultation or treatments by a physician, physiotherapist, or chiropractor in the previous 12 months?**

-  No
-  Yes

**55. Have you ever had sleeping difficulties because of the low back pain?**

-  No
-  Yes, how often? _________________

**56. Have you ever had low back pain that radiates to your lower extremity/ies?** You can choose more than one option.

-  No
-  Yes, to buttocks
-  Yes, to thigh
-  Yes, to knee
-  Yes, to shin, calf or foot

**57. Have you ever had surgery because of low back pain?**

-  No
-  Yes

**58. How did your low back pain start?**

-  Suddenly after a back injury
-  Over time, without an injury
-  Both

**59. Do the symptoms present themselves during some specific training or sport?**

-  No, not in any specific situation
-  Yes, during my own sport/hobby
-  Yes, during strength training
-  Yes, during plyometric training (jumps and hops)
-  Yes, in some other situation. What? ________________________________

**60. How many days of school have you missed due to low back pain in the previous 12 months?**

-  0 days
-  1-7 days
-  8-30 days
-  Over 30 days

**61. How many days have you had to take a break from playing sports or competitions/games due to low back pain in the previous 12 months?**

-  0 days
-  1-7 days
-  8-30 days
-  Over 30 days

**62. Have you used nonsteroidal anti-inflammatory drugs (NSAIDs) or pain killers for your low back?**

-  No

☐ Yes

**How often and with what dose did you take NSAIDs or pain killers** (e.g. Burana 400mgx3, longer treatment, daily, weekly ) and **who advised you to take it?** (e.g. my self, coach, doctor, dad)

Medication and dose: ___________________
How often: ____________________________
Who advised to use: ______________________

**THANK YOU FOR YOUR ANSWERS!**
